# Determination of Inorganic Elements in the Rhizome of *Paris polyphylla* Smith Var. *chinensis* (Franch.) Hara by Using Inductively Coupled Plasma Mass Spectrometry

**DOI:** 10.1155/2019/4946192

**Published:** 2019-06-09

**Authors:** Tiezhu Chen, Juan Lin, Jun Yang, Yina Tang, Chunmei Zhang, Tao Zhang, Feiyan Wen, Qingmao Fang, Hao Zhang

**Affiliations:** ^1^West China School of Pharmacy, Sichuan University, Chengdu 610041, China; ^2^Sichuan Provincial Key Laboratory of Quality and Innovation Research of Chinese Materia Medica, Sichuan Academy of Chinese Medicine Sciences, Chengdu 610041, China

## Abstract

The objective of this study was to investigate the concentrations of inorganic elements in the rhizome of *Paris polyphylla* Smith var. *chinensis* (Franch.) Hara of different planting years and cultivation conditions. Twenty-five inorganic elements including Al, As, B, Ba, Be, Bi, Ca, Cd, Co, Cr, Cu, Fe, K, Li, Mg, Mn, Na, Ni, P, Pb, Se, Sr, Ti, V, and Zn in the rhizome were determined by using inductively coupled plasma mass spectrometry (ICP-MS). The analytical method was validated by measuring several parameters including linearity, correlation coefficient, limit of detection (LOD), limit of quantification (LOQ), and recovery. The linear working ranges were three, 0–300 *μ*g/L, 0–500 *μ*g/L, and 0–1000 *μ*g/L, and the correlation coefficients (*r*) values were higher than 0.998. LOD varied from 0.001 *μ*g/L (Be) to 11.957 *μ*g/L (P), and LOQ varied from 0.003 *μ*g/L (Be) to 35.870 *μ*g/L (P). The recoveries spanned from 95.2 (Co) to 105.3% (Pb). Validation parameters showed the possibility of using whole of the sample preparation procedures used in this study. Based on the determined results, it is indicated that the toxic elements As, Cd, and Pb had no ingestion risk. The planting years and cultivation conditions had significant effects on the concentrations of inorganic elements of *P*. *polyphylla* var. *chinensis*. The concentrations of inorganic elements in cultivated samples were different from those in wild samples. The results can provide useful theoretical basis for the quality control and rational use of *P*. *polyphylla* var. *chinensis*.

## 1. Introduction

*Paris polyphylla* Smith var. *chinensis* (Franch.) Hara (PPC) is widely cultivated in China, and its dried rhizome is the well-known Chinese herb Rhizoma Paridis. It is often used as a medicinal material in Traditional Chinese Medicine. In addition, it is also used for medicinal purposes by some ethnic minorities in China, such as Tibetan and Yi in Sichuan Province and Dai in Yunnan Province. Rhizoma Paridis has many medicinal effects. It can clear away heat and expel evils, eliminate swelling and relieve pains, and cool the liver and arrest convulsion. In clinical practice, it can be used for the treatment of hard furuncle and carbuncle, sore throat, snake bite and insect sting, traumatic wound, and convulsions [1]. PPC belongs to Rhizoma Paridis of Liliaceae and is distributed in tropical to temperate regions of Eurasia [2], especially in Southwestern China. The main chemical component is polyphyllin [3]. In addition to hemostatic, analgesic, sedative, antibacterial, spermicidal, and immunomodulatory activities, polyphyllin also has antitumor effect [4–6]. Currently, scholars from various countries have reported a number of antitumor studies on this compound [7–9].

In recent years, numerous studies have found that the efficacy of Traditional Chinese Medicine is not only related to the organic components, but also closely related to the types and concentrations of inorganic elements [10–12]. In addition to the essential roles in the participation and regulation of metabolism, inorganic elements also represent an important factor for the exertion of pharmacological effects [13–16]. Furthermore, they play important roles in eliminating disease and improving immunity. However, some of the inorganic elements such as As, Cd, and Pb are harmful and can cause toxic effects in excessive amount. Both WHO and China's Ministry of Foreign Trade and Economic Cooperation have developed the limit standards for harmful elements in Chinese herbal medicines. The toxicity limits of As, Cd, and Pb recommended by the WHO are 5.0, 0.3, and 10.0 *μ*g/g, respectively [17], while the toxicity limits of As, Cd, and Pb recommended by the Chinese “Green Trade Standards of Importing & Exporting Medicinal Plants & Preparations” are 2.0, 1.0, and 10.0 *μ*g/g, respectively [18]. Therefore, the determination and analysis of inorganic elements have important significance not only for the efficacy and safety evaluation of Traditional Chinese Medicine, but also for the establishment of harmful elements limit. However, the evaluation of inorganic elements in PPC is unsatisfactory [19, 20], which hinders the research and application of this product. Therefore, the determination of inorganic element concentrations in PPC has important significance for the systematic study of pharmacological effects.

The concentrations of inorganic elements in the rhizomes of PPC with different planting years were determined by using inductively coupled plasma mass spectrometry (ICP-MS), followed by statistical analysis of the measured concentration data, including correlation analysis (CA), principal component analysis (PCA), and cluster analysis (HCA). Meanwhile, the effects of different cultivation conditions (propagation mode, altitude, soil properties, and harvesting time) on the concentrations of inorganic elements were further studied, so as to provide certain theoretical basis for the quality control and rational use of PPC.

## 2. Materials and Methods

### 2.1. Collection and Preparation of Plant Samples

The samples of PPC of different planting years and cultivation conditions were all collected from Pengzhou of Chengdu City, Sichuan, China. The rhizome of wild PPC was also collected from Pengzhou. The number of samples of 1-year plants, 2- to 8-year cultivated plants, and wild plants were 60, 30, and 30, respectively, while the number of samples used for different cultivation parameters were 20. All samples were *Paris polyphylla* Smith var. *chinensis* (Franch.) Hara, as identified by Professor Zhang Hao from Sichuan University. Similar planting-age or wild-type samples were first washed with tap water and rinsed with ultrapure deionized water, then dried at 50°C for 72 h and uniformly pulverized with a pulverizer. Three randomized samplings were performed for repeated measurement.

### 2.2. Reagents

Nitric acid 67% (Kelong Chemical Reagent, China) and hydrogen peroxide 30% (Kelong Chemical Reagent, China) used for digestion were of analytical purity. Internal standard elements, consisting of 1000 mg·L^−1^ of ^6^Li, ^45^Sc, ^73^Ge, ^115^In, and ^185^Re, were obtained from Thermo Fisher Scientific (USA). Calibration solutions of 1000 mg·L^−1^ of Al, As, B, Ba, Be, Bi, Ca, Cd, Co, Cr, Cu, Fe, K, Li, Mg, Mn, Na, Ni, P, Pb, Se, Sr, Ti, V, and Zn were all purchased from China National Analysis Center for Iron and Steel. The certified reference material used for internal quality control was Citrus leaf reference material (CRM, GBW10020) from the Institute of Geophysical and Geochemical Exploration, China, while, all other chemicals were of analytical grade. Internal standard elements of ^6^Li, ^45^Sc, ^73^Ge, ^115^In, and ^185^Re were used as mixed internal standards (200 *μ*g/L). The calibration standard solutions (0–100 *μ*g/L) were prepared by diluting mixed stock standards (10 mg/L) with 4% (V/V) nitric acid solution appropriately. All experimental solutions were prepared with ultrapure water (18.2 MΩ·cm^−1^), which was produced by a purification system (Milli-Q Gradient, Millipore, USA).

### 2.3. Instruments Setting and Optimization

The samples were processed in microwave digestion apparatus (WX-8000, Preekem Co., China) before digestion analysis; see Table 1 for specific operating conditions. Elements in the samples, including Al, As, B, Ba, Be, Bi, Ca, Cd, Co, Cr, Cu, Fe, K, Li, Mg, Mn, Na, Ni, P, Pb, Se, Sr, Ti, V, and Zn, were determined with ICP-MS (ICAP Q, Thermo Scientific, USA). The operation conditions of ICP-MS are shown in Table 2.

### 2.4. Digestion of the Samples

Powdered samples (200 mg) dried to constant weight were precisely weighed and placed in a polytetrafluoroethylene (PTFE) digestion tank soaked with 10% (V/V) nitric acid. After incubation with 7 mL nitric acid and 1 mL hydrogen peroxide for 1 h, the samples were sealed and digested in microwave. The digestion followed the working procedures of microwave digestion. After the temperature decreased to below 80°C, the smoke was dispersed, and the samples were removed and placed in an electric heating sleeve to expel the acid to about 1 mL. Then, the samples were transferred into a 25 mL volumetric flask after microwave digestion with deionized water. Later, the samples were transferred into another 25 mL volumetric flask after washing three times with deionized water and metered to volume. Finally, the samples were filtered with membrane after mixing evenly to serve as test solution. Three blank solutions were also prepared and digested in the same manner.

### 2.5. Statistical Analysis

Statistical analysis was performed on the citrus leaves reference materials (CRM, GBW10020) using *t*-test in Excel. SPSS version 21 was used for statistical analysis (CA, PCA, and HCA). In the HCA process, all data were normalized to 1, and the Chi-square method was used for cluster analysis.

### 2.6. Effects of Propagation Mode, Altitude, Soil Properties, and Harvesting Time

In the same plot, the seedlings, multiple shoots, and rhizome cuts with buds were cultivated to study the effects of propagation methods. To study the effect of altitude, rhizome cuts with buds were cultivated at altitudes of 500 m, 1000 m, and 1500 m, respectively. Rhizome cuts with buds were cultivated in deep and loose layers of raw soil, mellow soil, loam soil, and humus soil, in order to study the impact of soil properties. Rhizome of the selected PPC, which has been cultivated for eight years using seedlings as propagation materials in the same plot, were collected in June, August, October, and December, respectively, to study the impact of harvesting time.

The determination of inorganic element concentrations in the samples obtained from the cultivation mentioned above was carried out according to the aforementioned method.

## 3. Results and Discussion

### 3.1. Analytical Characteristics

The overall parameters of the proposed method for inorganic element analysis were obtained and are listed in Table 3.

The linear ranges were beyond at least 5 calibration points, which were separated into three intervals, including 0–300 *μ*g/L, 0–500 *μ*g/L, and 0–1000 *μ*g/L. The correlation coefficients (*r*) values were higher than 0.998 [21] for whole elements of the analytes, and the regression analysis showed that the linear correlation between concentration and signal intensity was significant (*p* < 0.05). LOD, defined as the concentration equivalent to three times the standard deviation of ten measurements of the reagent blank, varied from 0.001 *μ*g/L (Be) to 11.957 *μ*g/L (P), while LOQ, defined as the concentration equivalent to ten times the standard deviation, varied from 0.003 *μ*g/L (Be) to 35.870 *μ*g/L (P). Recoveries spanned from 95.2 (Co) to 105.3% (Pb), indicating that the determination results of all the elements were within the range of 80%–120% recommended by FDA [22]. The result highlights the possibility of using whole of the sample preparation procedures in this study.

The accuracy and precision of this method were validated using certified reference materials of citrus leaves (CRM, GBW10020), and the certified value versus the obtained values by using ICP-MS is given in Table 4. There was no significant statistical difference between the obtained value and the certified value (*p* < 0.05), indicating a good consistency between the obtained value and the certified value. Therefore, the accuracy and precision of the present method seems acceptable.

### 3.2. Inorganic Element Concentrations in the Rhizomes of PPC

The rhizome of PPC was not only abundant in Ca, K, Mg, Mn, Na, and P, but also rich in B, Ba, Cr, Cu, Fe, Mn, Ni, Sr, and Zn, regardless of the planting years. In addition, it contained not only essential trace elements such as Se and V, but also toxic elements such as As, Cd, and Pb, even though the concentrations of these toxic elements were very low (Table 5). For example, although the concentration of As was the highest in the first year samples, reaching 1.32 *μ*g/g, it was still lower than the minimal limit of 5.0 *μ*g/g as defined by the World Health Organization [17, 23] and also lower than the minimal limit of 2.0 *μ*g/g according to “Green Industry Standard for Import and Export of Medicinal Plants and Preparations” defined by the Ministry of Foreign Trade and Cooperation of China [18]. Under normal circumstances, the rhizome of PPC cultivated for 1 year was too small in size to be used for medicinal purposes. Therefore, the consumption of PPC rhizome was unlikely to cause As ingestion risk. The acceptable daily intake (ADI) of Cd, another toxic element detected in all samples, was 70 *μ*g/d [24, 25]. Even in the first-year samples with the highest Cd concentration, the value was merely 0.50 *μ*g/g. In the “Chinese Pharmacopoeia,” the recommended intake of Rhizoma Paridis was 3–9 g/d [1]. If calculated by the median 6 g/d, the maximal total intake of Cd was 3.0 *μ*g/d. Therefore, there was no Cd ingestion risk, either. In addition, the measured Pb concentration was (0.35–8.02 *μ*g/g). When compared with the ADI of 214 *μ*g/d Pb as specified by the Food and Agriculture Organization/World Health Organization [26], the risk of Pb was relatively low. Bi and Ti were not detected in all samples.

**Figure 1:**
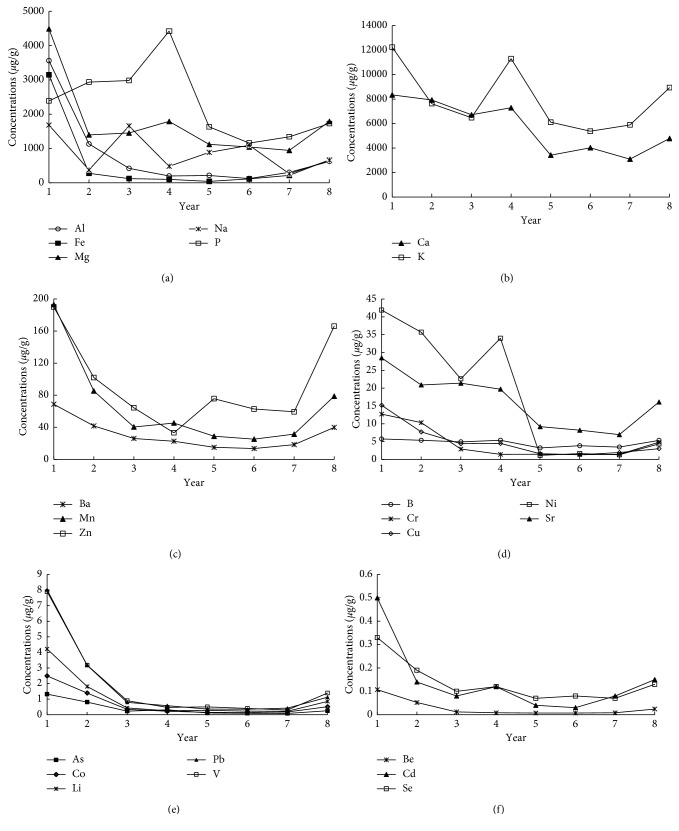
Variation curves of inorganic elements of different planting years of PPC.

The planting years affected the content of inorganic elements in the rhizome of PPC. The variation curves of inorganic elements in samples with different planting years are shown in Figure 1. The changing trends in the content of Al (Figure 1(a)), Ba (Figure 1(c)), Cr (Figure 1(d)), As, Co, Fe, Li, Pb, and V (Figure 1(e)) are consistent in the samples with different growth years. In all cases, the contents were the highest in Year 1 and declined in Year 2. In Years 3–7, the changing trends of contents in the samples were not significant. Then, the contents increased in Year 8. The content of Cd (Figure 1(f)), K (Figure 1(b)), Mg (Figure 1(a)), Ni (Figure 1(d)), and Se (Figure 1(f)) in raw samples declined gradually in Years 1–3 and increased slightly in Year 4. After the sample concentration curves became stable in Years 5-6, the contents increased gradually in Years 7-8. The changing trends in contents of remaining elements were inconsistent with the changes of the above elements.

Among samples of all planting years, the elements Al, As, B, Ba, Be, Ca, Cd, Co, Cr, Cu, Fe, K, Li, Mg, Mn, Na, and Ni in Year 1 samples were the highest. The contents of majority inorganic elements were lower in Years 2–6 samples than in Year 1 samples. However, the contents of inorganic elements were slightly higher in Year 8 samples than in Year 7 samples. The reasons for above changes may lie in the accumulation of starch and other substances in the rhizome of PPC. In Year 1, the contents of starch and other substances were the lowest, resulting in the highest contents of inorganic elements. With planting years increasing, the accumulation of starch and other substances increased significantly, resulting in a gradual decline in the percentage of inorganic elements. Year 8 is another accelerating growth period for the rhizome of PPC. The folds of rhizome dry weight gain decreased [27], which slowed down the growth rate of starch and other substances, and then led to an elevation of the percentage of inorganic elements in Year 8.

### 3.3. Correlation Analysis

Correlation analysis (CA) was used to analyze the correlation of inorganic elements in PPC of different planting years. See Table S1 in the data for the correlation matrix analysis. The correlation coefficient of toxic element As with Al, Ba, Be, Cd, Co, Cr, Cu, Fe, Li, Mg, Mn, Pb, Se, and V was positive and close to 1, indicating a strong and positive correlation of As with Al, Ba, Be, Cd, Co, Cr, Cu, Fe, Li, Mg, Mn, Pb, Se, and V. It could be referred that these elements in the plants may have a certain synergistic effect during absorption and accumulation, consistent with the findings of Zhang's study on the nutrient elements of Paridis Rhizoma cultivated in Three Gorges Reservoir Region [20]. However, the relevant mechanisms are yet to be studied.

### 3.4. Principal Component Analysis

Principal component analysis (PCA) was conducted to determine the inorganic elements in PPC of different planting years. See Table S2 in the data for the scores of the principal components. For the first two principal components with characteristic root >1, their cumulative contribution rate was 90.32%, suggesting that these components played a decisive role. Contribution of the first principal component was 78.52%, and Al, As, Ba, Be, Cd, Co, Cr, Cu, Fe, Li, Mg, Mn, Pb, Se, and V were the primary variables of the first principal component. Contribution of the second principal component was 11.80%, and P predominated in the second principal component. Based on the loadings, the characteristic elements of PPC included Al, As, B, Ba, Be, Cd, Co, Cr, Cu, Fe, Li, Mg, Mn, P, Pb, Se, and V.

**Figure 2:**
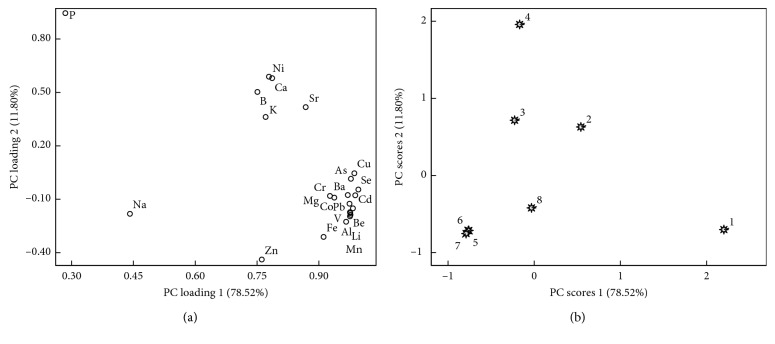
Varimax rotated principal component (a) loadings and (b) scores of different planting years of PPC.

The first two principal component loadings and scores of inorganic elements in PPC of different planting years are presented in Figure 2. It can be clearly seen from Figure 2(a) that Al, As, Ba, Be, Cd, Co, Cr, Cu, Fe, Li, Mg, Mn, Pb, Se, and V were clustered together, indicating the similarities among these elements. As shown in Figure 2(b), the distributions of samples collected in Years 1–4 and 8 were relatively scattered, while the samples collected in Years 5–7 were aggregated, suggesting they had similar elemental patterns and were closely related. PCA results clearly showed that the samples of PPC with different planting years varied from the elemental perspectives, which might affect the quality and pharmacological properties. Previously, the sample quality of PPC in Years 5–7 was determined, and the similarity was observed [27]. In the meantime, some literature studies showed that the inorganic elements in Rhizoma Paridis were closely related to polyphyllin. For instance, Ni and Sr were positively correlated with total polyphyllin I, II, VI, and VII, while Fe was negatively correlated with total polyphyllin I, II, VI, and VII [28]. The inorganic elements in Rhizoma Paridis also had certain correlation with various pharmacological activities of Rhizoma Paridis. Ca and Zn were related to the pharmacological effects of anti-inflammation, hemostasis, and sarcogenesis; Mg was related to the pharmacological effects of immunomodulation and detoxification and could be used for the treatment of hard furuncle, carbuncle, and swelling; Zn was related to the antibacterial and antiviral effects of Rhizoma Paridis [19, 29–31]. Future studies should mainly focus on the assessment of pharmacological effects of these samples in given years. If the pharmacological effects are similar, the planting years of PPC can be adjusted to reduce the cultivation cost.

### 3.5. Cluster Analysis

**Figure 3:**
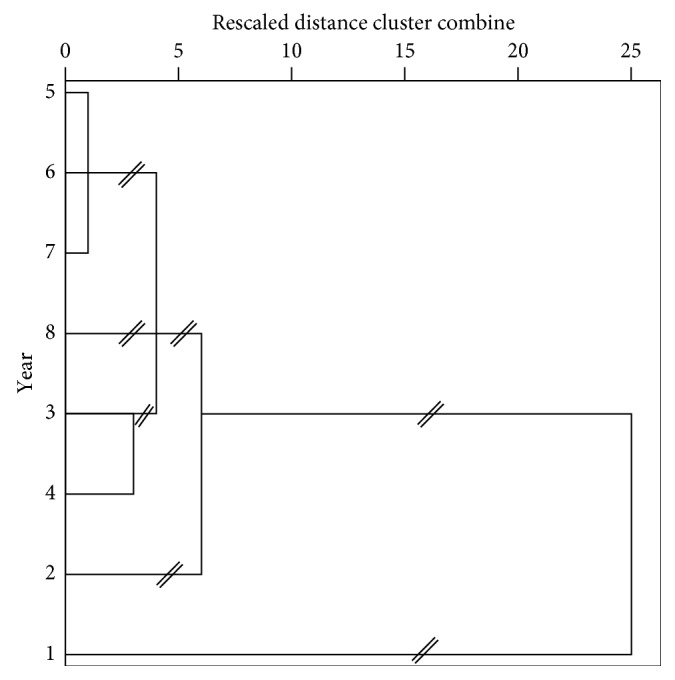
Dendrogram of hierarchical cluster analysis for PPC samples collected in different planting years.

Hierarchical cluster analysis (HCA) was used to further analyze PPC of different planting years (Figure 3). Samples collected in Year 1 were alone without companion, significantly different from those collected in other years. Samples collected in Years 3 and 4 were included in the same group, indicating a close correlation between them. Samples collected in Years 5–7 were assigned in another group, which was consistent with PCA results (samples planted for 5–7 years belong to the same group). It usually takes 5–8 years from the transplant of seedlings to herb harvesting. According to HCA and PCA, the elemental patterns of samples in Years 5–7 were similar, but they significantly differed from those of samples collected in Year 8. The difference was mainly due to the elevation of trace element concentrations in Year 8 samples. The effects of different element concentrations on the quality were unknown. As is known, the quality evaluation of Chinese medicinal materials is not limited to inorganic elements, so the main effective components should also be evaluated. Our previous study found that the amount of active ingredients of Polyphyllin I, II, VI, and VII in 5- to 8-year planting year *Paris polyphylla* increases with the planting years [27], and therefore, the recommended planting period is 7 years.

### 3.6. Cultivation Parameters

**Figure 4:**
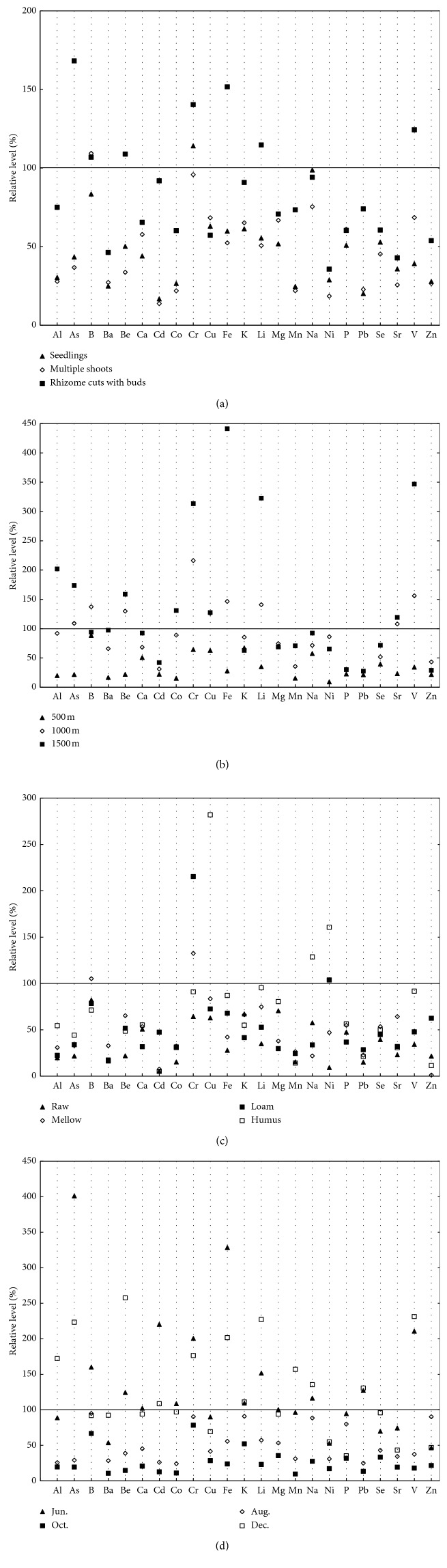
Influence of (a) propagation mode, (b) altitude, (c) soil property, and (d) harvesting time on element concentrations in the rhizomes of PPC.

With the spreading of cultivated PPC in Southwestern China, researches on the cultivation should be deepened. Therefore, this study also determined the concentrations of inorganic elements in the rhizomes of PPC under different conditions (propagation mode, altitude, soil properties, and harvesting time). See Table S3 in the data for the results. To calculate the relative level, concentrations of inorganic elements in cultivated samples were compared with those in wild samples [32], and relative level = [inorganic element content of cultivated sample/inorganic element content of wild sample] × 100%. The calculated results are shown in Figure 4.

As shown in Figure 4(a), compared with wild samples, only Cr element concentration increased in samples cultivated with seedling, and only B element concentration increased in samples cultivated with multiple shoots, while all the remaining element concentrations in samples cultivated with seedling and multiple shoots decreased somewhat. In cultivated samples using rhizome cuts with buds, the concentrations of As, B, Be, Cr, Fe, Mn, Na, Pb, Li, and V increased, while the concentrations of the remaining elements decreased.

For the impact of altitude (Figure 4(b)), all elemental concentrations were lower in samples collected at 500 m altitude than those in wild samples. However, as the altitude increased, the concentrations of the corresponding elements increased. Except for B, Ni, and Zn, the concentrations of all elements were higher in samples collected at 1500 m altitude than those in samples collected at 1000 m altitude.

As shown in Figure 4(c), the concentrations of all elements in the samples cultivated in raw soil decreased. In samples cultivated in mellow soil, the concentrations of B and Cr increased, while concentrations of the others decreased. Except for Cr and Ni, the concentrations of the elements were lower in samples cultivated in loam soil than those in wild samples. With comparison of other types of soil, the concentration of Cu and Ni were the highest in samples cultivated in humus soil, while the concentrations of other elements were lower than those in wild samples.

In the present study, on the impact of harvesting time (Figure 4(d)), the concentrations of all elements except Ni, Se, Sr, and Zn in samples harvested in June were close to or higher than those in wild samples. The concentrations of all elements were lower in samples harvested in August and October than those in wild samples, and the concentrations of elements in samples harvested in October were the lowest. Except for Al, As, Be, Cd, Cr, Fe, K, Li, Mn, Na, Pb, and V, the concentrations of remaining elements were lower in samples harvested in December than those in wild samples.

## 4. Conclusion

In this study, ICP-MS was used to determine the concentrations of inorganic elements in the rhizomes of PPC with different planting years or under different cultivating conditions. The results show that the rhizome of PPC contains numerous elements, including Al, As, B, Ba, Be, Ca, Cd, Co, Cr, Cu, Fe, K, Li, Mg, Mn, Na, Ni, P, Pb, Se, Sr, V, and Zn. Planting years have significant effects on the concentrations of inorganic elements. The elemental patterns of element concentrations in samples collected in different planting years are different. The elemental patterns are similar in samples collected in Years 5–7, but significantly different from samples collected in Year 8. In addition, propagation mode, altitude, soil properties, and harvesting time also have certain effects on the concentrations of these elements in the rhizome of PPC. When cultivated PPC is compared with wild ones, the concentrations of inorganic elements are lower in most cultivated samples than those in wild samples, which can explain to some extent why a better medicinal effect is observed with wild PPC. In future studies, it is expected to unravel the relationship between inorganic elements and pharmacological actions of PPC.

## Figures and Tables

**Table 1:** Microwave operating conditions for the digestion of PPC.

Step	*T* (°C)	Pressure (atm)	Power (W)	Holding time (min)
1	120	40	1600	10
2	150	40	1600	20
3	180	40	1600	20

**Table 2:** Optimum ICP-MS operating conditions for the analysis of PPC.

Instrument parameter	Condition
Radio frequency power	1.4 kW
Spray chamber temperature	2.7°C
Nebulizer pump	0.1 rps
Cool flow	13 L/min
Auxiliary flow	0.8 L/min
Plasma flow	13 L/min
Nebulizer	0.88/min
Sampling depth	5 mm
No. of replicates per sample	3
Isotopes measured	^6^Li^*∗*^, ^7^Li, ^9^Be, ^11^B, ^23^Na, ^24^Mg, ^27^Al, ^31^P, ^39^K, ^44^Ca, ^45^Sc^*∗*^, ^48^Ti, ^51^V, ^52^Cr, ^55^Mn, ^57^Fe, ^59^Co, ^60^Ni, ^63^Cu, ^66^Zn, ^73^Ge^*∗*^, ^75^As, ^77^Se, ^88^Sr, ^111^Cd, ^115^In^*∗*^, ^137^Ba, ^185^Re^*∗*^, ^208^Pb, ^209^Bi

^*∗*^Internal standards.

**Table 3:** Analytical parameters of the ICP-MS method.

Element	Linear range (*μ*g/L)	Regression equation	Correlation coefficient (*r*)	Limit of detection (*μ*g/L)	Limit of quantification (*μ*g/L)	Recovery (%)
Al	0–1000	*y* = 73466.319*x* + 1701428.801	0.9994	2.646	7.937	101.5
As	0–500	*y* = 13044.717*x* + 1069.046	0.9990	0.006	0.021	95.7
B	0–500	*y* = 11565.476*x* + 22820.933	0.9999	0.011	0.033	99.7
Ba	0–1000	*y* = 25039.481*x* + 1050274.760	0.9988	0.005	0.016	96.8
Be	0–500	*y* = 8883.222*x*	0.9997	0.001	0.003	99.4
Bi	0–1000	*y* = 149495.303*x* + 1748444.689	0.9996	0.003	0.010	101.4
Ca	0–1000	*y* = 6576.577*x* + 233419.741	0.9982	3.770	11.309	96.8
Cd	0–500	*y* = 19752.118*x* + 185.002	0.9992	0.008	0.027	99.1
Co	0–500	*y* = 82931.850*x* + 1665.120	0.9983	0.003	0.010	95.2
Cr	0–500	*y* = 63498.325*x* + 111919.636	0.9992	0.116	0.354	96.8
Cu	0–300	*y* = 60906.536*x* + 50216.325	0.9999	0.203	0.613	97.9
Fe	0–1000	*y* = 4721.293*x* + 363108.369	0.9993	1.923	5.769	100.4
K	0–1000	*y* = 98631.432*x* + 8811015.191	0.9988	3.618	10.867	105.1
Li	0–500	*y* = 29816.579*x* + 525.012	0.9985	0.008	0.025	98.6
Mg	0–1000	*y* = 54373.476*x* + 1003073.381	0.9994	0.717	2.152	101.2
Mn	0–1000	*y* = 155163.046*x* + 1627478.220	0.9996	0.008	0.025	99.5
Na	0–1000	*y* = 98631.432*x* + 8811015.191	0.9987	0.049	0.148	96.7
Ni	0–500	*y* = 24227.985*x* + 3540.534	0.9989	0.102	0.305	97.9
P	0–1000	*y* = 5417.667*x* + 516280.862	0.9998	11.957	35.870	99.4
Pb	0–500	*y* = 87958.918*x* + 44905.734	0.9983	0.063	0.194	105.3
Se	0–500	*y* = 1225.718*x* + 1690.126	0.9993	0.450	1.358	100.2
Sr	0–500	*y* = 131220.830*x* + 10289.297	0.9983	0.027	0.085	103.4
Ti	0–500	*y* = 84686.312*x* + 989169.607	0.9987	0.056	0.168	98.1
V	0–500	*y* = 68466.636*x* + 33570.526	0.9985	0.007	0.022	104.3
Zn	0–500	*y* = 23715.100*x* + 137716.819	0.9989	0.031	0.096	101.7

**Table 4:** Accuracy assessment by analysis of the citrus leaves certified reference material (CRM, GBW10020; *n* = 3).

Element	Certified value (*μ*g/g)	Obtained value (*μ*g/g)
Al	1150 (8.7%)^a^	1090 (7.3%)^a^
As	1.1 (18.2%)^a^	1.0 (20%)^a^
B	32 (9.4%)^a^	31 (16.1%)^a^
Ba	98 (6.1%)^a^	96 (9.4%)^a^
Be	0.031 (22.6%)^a^	0.033 (18.2%)^a^
Bi	0.23 (10.9%)^a^	0.228 (11.1%)^a^
Ca	42000 (9.5%)^a^	41800 (9.7%)^a^
Cd	0.17 (11.8%)^a^	0.16 (18.8%)^a^
Co	0.23 (26.1%)^a^	0.24 (33.3%)^a^
Cr	1.25 (8.8%)^a^	1.27 (8.7%)^a^
Cu	6.6 (7.6%)^a^	6.4 (10.9%)^a^
Fe	480 (6.3%)^a^	492 (11.4%)^a^
K	7700 (5.2%)^a^	7920 (8.2%)^a^
Li	1.0 (10%)^a^	0.9 (11.1%)^a^
Mg	2340 (3%)^a^	2280 (7.1%)^a^
Mn	30.5 (4.9%)^a^	30.1 (14%)^a^
Na	130 (15.4%)^a^	124 (25%)^a^
Ni	1.1^a,*∗*^	1.0 (10%)^a^
P	1250 (7.2%)^a^	1220 (9%)^a^
Pb	9.7 (9.3%)^a^	9.5 (8.4%)^a^
Se	0.17 (17.6%)^a^	0.18 (27.8%)^a^
Sr	170 (5.9%)^a^	175 (13.7%)^a^
Ti	38 (26.3%)^a^	40 (22.5%)^a^
V	1.16 (11.2%)^a^	1.12 (25%)^a^
Zn	18 (11.1%)^a^	19 (15.7%)^a^

% differences between the theoretical and experimental values are given in brackets. ^a^Values within each line followed by the same character is not statistically different (*p* < 0.05). ^*∗*^Reference value.

**Table 5:** Element concentrations in the rhizomes of different planting years of PPC (*n* = 3; mean ± SD).

Element	1st year (*µ*g/g)	2nd year (*µ*g/g)	3rd year (*µ*g/g)	4th year (*µ*g/g)	5th year (*µ*g/g)	6th year (*µ*g/g)	7th year (*µ*g/g)	8th year (*µ*g/g)
Al	3561 ± 22	1131 ± 31	419 ± 12	197 ± 8	213 ± 11	123 ± 10	303 ± 9	621 ± 20
As	1.32 ± 0.13	0.81 ± 0.08	0.23 ± 0.02	0.31 ± 0.01	0.11 ± 0.02	0.09 ± 0.01	0.08 ± 0.01	0.25 ± 0.02
B	5.75 ± 0.41	5.37 ± 0.25	4.94 ± 0.34	5.38 ± 0.19	3.23 ± 0.11	3.85 ± 0.15	3.48 ± 0.24	5.36 ± 0.27
Ba	68.8 ± 0.7	41.8 ± 0.5	26.1 ± 0.2	22.7 ± 0.3	15.1 ± 0.2	13.6 ± 0.2	18.5 ± 0.1	39.9 ± 0.2
Be	0.107 ± 0.002	0.052 ± 0.002	0.012 ± 0.001	0.008 ± 0.001	0.007 ± 0.001	0.007 ± 0.001	0.008 ± 0.001	0.024 ± 0.003
Bi	<LOQ	<LOQ	<LOQ	<LOQ	<LOQ	<LOQ	<LOQ	<LOQ
Ca	8339 ± 60	7924 ± 22	6717 ± 35	7291 ± 50	3419 ± 40	4026 ± 40	3094 ± 35	4782 ± 50
Cd	0.50 ± 0.02	0.14 ± 0.01	0.08 ± 0.01	0.12 ± 0.01	0.04 ± 0.00	0.03 ± 0.00	0.08 ± 0.01	0.15 ± 0.02
Co	2.49 ± 0.07	1.39 ± 0.05	0.34 ± 0.03	0.22 ± 0.02	0.15 ± 0.01	0.14 ± 0.01	0.17 ± 0.01	0.51 ± 0.03
Cr	12.72 ± 0.44	10.34 ± 0.23	2.92 ± 0.21	1.42 ± 0.14	1.45 ± 0.12	1.33 ± 0.09	1.42 ± 0.08	4.84 ± 0.16
Cu	15.2 ± 0.21	7.74 ± 0.34	4.41 ± 0.28	4.51 ± 0.29	1.62 ± 0.03	1.33 ± 0.11	1.93 ± 0.16	3.03 ± 0.14
Fe	3149 ± 42	277 ± 30	122 ± 8	100 ± 5	40 ± 3	112 ± 5	214 ± 7	666 ± 15
K	12234 ± 80	7621 ± 68	6476 ± 45	11291 ± 52	6111 ± 50	5375 ± 47	5890 ± 37	8929 ± 45
Li	4.22 ± 0.20	1.81 ± 0.13	0.43 ± 0.03	0.25 ± 0.01	0.28 ± 0.02	0.23 ± 0.02	0.22 ± 0.03	0.85 ± 0.07
Mg	4489 ± 50	1396 ± 32	1451 ± 44	1790 ± 38	1120 ± 42	1042 ± 51	944 ± 36	1789 ± 44
Mn	193 ± 4	85.3 ± 2.3	40.5 ± 1.7	45.4 ± 1.5	28.9 ± 1.1	25.3 ± 1.4	31.5 ± 0.9	78.8 ± 1.4
Na	1683 ± 54	374 ± 36	1658 ± 26	481 ± 50	886 ± 13	1094 ± 31	263 ± 11	656 ± 10
Ni	41.9 ± 0.6	35.7 ± 0.7	22.6 ± 0.4	34.0 ± 0.3	1.15 ± 0.13	1.65 ± 0.16	1.30 ± 0.14	4.36 ± 0.24
P	2387 ± 33	2937 ± 27	2983 ± 50	4422 ± 41	1631 ± 29	1157 ± 18	1341 ± 34	1727 ± 24
Pb	8.02 ± 0.25	3.17 ± 0.04	0.79 ± 0.02	0.58 ± 0.02	0.36 ± 0.01	0.35 ± 0.01	0.42 ± 0.01	1.12 ± 0.03
Se	0.33 ± 0.02	0.19 ± 0.01	0.10 ± 0.01	0.12 ± 0.01	0.07 ± 0.01	0.08 ± 0.01	0.07 ± 0.01	0.13 ± 0.01
Sr	28.5 ± 0.4	20.9 ± 0.5	21.4 ± 0.5	19.7 ± 0.3	9.2 ± 0.4	8.21 ± 0.08	6.96 ± 0.19	16.1 ± 0.8
Ti	<LOQ	<LOQ	<LOQ	<LOQ	<LOQ	<LOQ	<LOQ	<LOQ
V	7.90 ± 0.07	3.18 ± 0.02	0.90 ± 0.01	0.47 ± 0.02	0.50 ± 0.02	0.40 ± 0.01	0.30 ± 0.02	1.39 ± 0.03
Zn	190 ± 4	102 ± 3	64.4 ± 2.9	33.2 ± 3.1	75.7 ± 4.6	62.8 ± 3.1	59.4 ± 1.1	166 ± 5

Bi and Ti concentrations were below the limit of quantification (LOQ).

## Data Availability

The data used to support the findings of this study are included within the article and the supplementary information file.

## Supplementary Materials

Supplementary Materials (1) Table S1 shows the correlation matrix for the inorganic elements concentrations in the rhizomes of different planting years of PPC. (2) Table S2 shows the varimax rotated factor loadings of the first two principal components obtained by principal component analysis of inorganic elements in the rhizomes of different planting years of *Paris polyphylla* Smith var. chinensis (Franch.) Hara (PPC). (3) Table S3 shows element concentrations in the rhizomes of propagation mode, altitude, soil property, and harvesting time of PPC by using inductively coupled plasma mass spectrometry (ICP-MS).
