# Supplementary material for: Determination of Inorganic Elements in the Rhizome of Paris polyphylla Smith Var. chinensis (Franch.) Hara by Using Inductively Coupled Plasma Mass Spectrometry
Source: J Anal Methods Chem. 2019 Jun 9;2019:4946192. doi: 10.1155/2019/4946192 (PMC6590584; doi:10.1155/2019/4946192)
Supplement: Supplementary Materials — (1) Table S1 shows the correlation matrix for the inorganic elements concentrations in the rhizomes of different planting years of PPC. (2) Table S2 shows the varimax rotated factor loadings of the first two principal components obtained by principal component analysis of inorganic elements in the rhizomes of different planting years of Paris polyphylla Smith var. chinensis (Franch.) Hara (PPC). (3) Table S3 shows element concentrations in the rhizomes of propagation mode, altitude, soil property, and harvesting time of PPC by using inductively coupled plasma mass spectrometry (ICP-MS). [file 4946192.f1.doc]

Table S1：Correlation matrix for the element concentrations in the rhizomes of different planting years of PPC.

|  | Al | As | B | Ba | Be | Ca | Cd | Co | Cr | Cu | Fe | K | Li | Mg | Mn | Na | Ni | P | Pb | Se | Sr | V | Zn |
| --- | --- | --- | --- | --- | --- | --- | --- | --- | --- | --- | --- | --- | --- | --- | --- | --- | --- | --- | --- | --- | --- | --- | --- |
| Al | 1.00 |  |  |  |  |  |  |  |  |  |  |  |  |  |  |  |  |  |  |  |  |  |  |
| As | **0.95** | 1.00 |  |  |  |  |  |  |  |  |  |  |  |  |  |  |  |  |  |  |  |  |  |
| B | 0.57 | 0.69 | 1.00 |  |  |  |  |  |  |  |  |  |  |  |  |  |  |  |  |  |  |  |  |
| Ba | **0.93** | **0.93** | **0.77** | 1.00 |  |  |  |  |  |  |  |  |  |  |  |  |  |  |  |  |  |  |  |
| Be | **0.98** | **0.98** | 0.62 | **0.96** | 1.00 |  |  |  |  |  |  |  |  |  |  |  |  |  |  |  |  |  |  |
| Ca | 0.62 | **0.80** | **0.88** | 0.71 | 0.67 | 1.00 |  |  |  |  |  |  |  |  |  |  |  |  |  |  |  |  |  |
| Cd | **0.98** | **0.98** | 0.68 | **0.96** | **0.99** | 0.72 | 1.00 |  |  |  |  |  |  |  |  |  |  |  |  |  |  |  |  |
| Co | **0.97** | **0.99** | 0.64 | **0.95** | **1.00** | 0.72 | **0.98** | 1.00 |  |  |  |  |  |  |  |  |  |  |  |  |  |  |  |
| Cr | **0.89** | **0.96** | 0.68 | **0.94** | **0.95** | 0.72 | **0.93** | **0.97** | 1.00 |  |  |  |  |  |  |  |  |  |  |  |  |  |  |
| Cu | **0.96** | **0.98** | 0.70 | **0.93** | **0.96** | **0.81** | **0.98** | **0.97** | **0.91** | 1.00 |  |  |  |  |  |  |  |  |  |  |  |  |  |
| Fe | **0.97** | **0.85** | 0.51 | **0.89** | **0.92** | 0.50 | **0.93** | **0.89** | **0.77** | **0.89** | 1.00 |  |  |  |  |  |  |  |  |  |  |  |  |
| K | 0.67 | 0.70 | **0.79** | 0.73 | 0.65 | 0.72 | **0.75** | 0.64 | 0.56 | 0.74 | 0.69 | 1.00 |  |  |  |  |  |  |  |  |  |  |  |
| Li | **0.99** | **0.97** | 0.60 | **0.95** | **1.00** | 0.66 | **0.99** | **0.99** | **0.94** | **0.96** | **0.94** | 0.66 | 1.00 |  |  |  |  |  |  |  |  |  |  |
| Mg | **0.94** | **0.87** | 0.64 | **0.89** | **0.90** | 0.64 | **0.93** | **0.87** | **0.75** | **0.92** | **0.97** | **0.82** | **0.92** | 1.00 |  |  |  |  |  |  |  |  |  |
| Mn | **0.98** | **0.95** | 0.68 | **0.98** | **0.98** | 0.67 | **0.99** | **0.96** | **0.91** | **0.95** | **0.96** | **0.75** | **0.98** | **0.95** | 1.00 |  |  |  |  |  |  |  |  |
| Na | 0.49 | 0.35 | 0.22 | 0.37 | 0.40 | 0.32 | 0.36 | 0.38 | 0.27 | 0.45 | 0.53 | 0.16 | 0.44 | 0.55 | 0.42 | 1.00 |  |  |  |  |  |  |  |
| Ni | 0.63 | 0.81 | **0.81** | 0.68 | 0.68 | **0.98** | 0.73 | 0.72 | 0.70 | **0.83** | 0.51 | 0.74 | 0.66 | 0.65 | 0.66 | 0.24 | 1.00 |  |  |  |  |  |  |
| P | 0.07 | 0.29 | 0.64 | 0.19 | 0.11 | **0.75** | 0.21 | 0.15 | 0.15 | 0.33 | -0.01 | 0.60 | 0.09 | 0.21 | 0.14 | -0.02 | **0.78** | 1.00 |  |  |  |  |  |
| Pb | **0.99** | **0.97** | 0.59 | **0.93** | **0.99** | 0.67 | **0.99** | **0.99** | **0.92** | **0.97** | **0.94** | 0.67 | **1.00** | **0.92** | **0.98** | 0.45 | 0.69 | 0.12 | 1.00 |  |  |  |  |
| Se | **0.97** | **0.98** | 0.72 | **0.96** | **0.98** | **0.76** | **0.99** | **0.98** | **0.93** | **0.98** | **0.92** | **0.76** | **0.98** | **0.94** | **0.99** | 0.42 | **0.75** | 0.24 | **0.98** | 1.00 |  |  |  |
| Sr | 0.73 | **0.82** | **0.91** | **0.83** | **0.75** | **0.94** | **0.80** | **0.77** | **0.76** | **0.86** | 0.66 | **0.78** | **0.75** | **0.78** | **0.79** | 0.47 | **0.90** | 0.64 | **0.75** | **0.83** | 1.00 |  |  |
| V | **0.99** | **0.97** | 0.60 | **0.94** | **1.00** | 0.67 | **0.98** | **0.99** | **0.93** | **0.97** | **0.94** | 0.66 | **1.00** | **0.92** | **0.98** | 0.47 | 0.67 | 0.11 | 1.00 | **0.98** | **0.76** | 1.00 |  |
| Zn | **0.79** | 0.69 | 0.52 | **0.87** | **0.80** | 0.32 | **0.76** | **0.76** | **0.77** | 0.66 | **0.81** | 0.50 | **0.80** | 0.74 | **0.85** | 0.34 | 0.24 | -0.22 | 0.76 | **0.76** | 0.53 | **0.78** | 1.00 |

Values in bold have high correlations with each other.

Table S2：Varimax rotated factor loadings of the first two principal components.

| Variable | Principal component | |
| --- | --- | --- |
|  | 1 | 2 |
| Al | **0.966** | -0.227 |
| As | **0.978** | 0.015 |
| B | 0.751 | 0.503 |
| Ba | **0.970** | -0.076 |
| Be | **0.976** | -0.178 |
| Ca | 0.787 | 0.580 |
| Cd | **0.988** | -0.078 |
| Co | **0.974** | -0.125 |
| Cr | **0.927** | -0.082 |
| Cu | **0.986** | 0.046 |
| Fe | **0.911** | -0.312 |
| K | 0.771 | 0.362 |
| Li | **0.976** | -0.196 |
| Mg | **0.938** | -0.091 |
| Mn | **0.983** | -0.151 |
| Na | 0.441 | -0.182 |
| Ni | 0.779 | 0.589 |
| P | 0.285 | **0.946** |
| Pb | **0.976** | -0.173 |
| Se | **0.996** | -0.045 |
| Sr | 0.868 | 0.417 |
| V | **0.977** | -0.188 |
| Zn | 0.761 | -0.439 |
| Explained variance (%) | 18.06 | 2.72 |
| Proportion of total variance (%) | 78.52 | 90.32 |

The bold values are the major contributors to each principal component.

Table S3：Influence of propagation mode, altitude, soil property, and harvesting time on the element concentrations in the rhizomes of PPC (n =3; mean ± SD).

|  | Propagation mode | | | Altitude | | | Soil properties | | | | Harvesting period | | | |
| --- | --- | --- | --- | --- | --- | --- | --- | --- | --- | --- | --- | --- | --- | --- |
| Element | Seedlings (*µg/g*) | Multiple shoots  (*µg/g*) | Rhizome cuts with buds (*µg/g*) | 500m  (*µg/g*) | 1000m (*µg/g*) | 1500m (*µg/g*) | Raw  (*µg/g*) | Mellow  (*µg/g*) | Loam  (*µg/g*) | Humus (*µg/g*) | Jun.  (*µg/g*) | Aug.  (*µg/g*) | Oct.  (*µg/g*) | Dec.  (*µg/g*) |
| Al | 315 ± 21 | 289 ± 16 | 779 ± 38 | 206 ± 19 | 956 ± 54 | 2102 ± 75 | 206 ± 31 | 321 ± 15 | 232 ± 7 | 566 ± 18 | 922 ± 31 | 267 ± 11 | 203 ± 6 | 1788 ± 45 |
| As | 0.13 ± 0.01 | 0.11 ± 0.01 | 0.52 ± 0.06 | 0.07 ± 0.01 | 0.34 ± 0.03 | 0.53 ± 0.02 | 0.07 ± 0.01 | 0.10 ± 0.01 | 0.10 ± 0.01 | 0.14 ± 0.02 | 1.23 ± 0.02 | 0.09 ± 0.01 | 0.07 ± 0.01 | 0.69 ± 0.05 |
| B | 3.98 ± 0.34 | 5.22 ± 0.21 | 5.10 ± 0.45 | 4.22 ± 0.19 | 6.56 ± 0.28 | 4.49 ± 0.61 | 3.92 ± 0.26 | 5.02 ± 0.35 | 3.74 ± 0.44 | 3.40 ± 0.37 | 7.63 ± 0.59 | 4.52 ± 0.41 | 3.18 ± 0.64 | 4.39 ± 0.22 |
| Ba | 21.8 ± 1.8 | 23.9 ± 2.4 | 40.6 ± 3.0 | 14.6 ± 2.1 | 57.8 ± 5.5 | 85.6 ± 7.4 | 14.1 ± 1.9 | 28.8 ± 4.6 | 15.7 ± 2.7 | 14.4 ± 2.0 | 47.3 ± 405 | 24.9 ± 1.8 | 9.23 ± 0.21 | 81.1 ± 5.2 |
| Be | 0.011 ±  0.001 | 0.008 ±  0.001 | 0.025 ±  0.003 | 0.005 ±  0.001 | 0.030 ±  0.006 | 0.036 ±  0.005 | 0.005 ±  0.001 | 0.015 ±  0.002 | 0.011 ±  0.002 | 0.011 ±  0.001 | 0.028 ±  0.004 | 0.009 ±  0.001 | 0.007 ±  0.001 | 0.059 ± 0.004 |
| Bi | < *LOQ* | < *LOQ* | < *LOQ* | < *LOQ* | < *LOQ* | < *LOQ* | < *LOQ* | < *LOQ* | < *LOQ* | < *LOQ* | < *LOQ* | < *LOQ* | < *LOQ* | < *LOQ* |
| Ca | 3830 ± 108 | 5023 ± 230 | 5701 ± 78 | 4417 ± 60 | 5952 ± 129 | 8061 ± 155 | 4417 ± 175 | 4665 ± 107 | 2768 ± 69 | 4812 ± 37 | 8896 ± 214 | 3935 ± 255 | 1801 ± 55 | 8172 ± 91 |
| Cd | 0.06 ± 0.01 | 0.40 ± 0.04 | 0.05 ± 0.01 | 0.78 ± 0.04 | 0.30 ± 0.05 | 0.40 ± 0.03 | 0.05 ± 0.01 | 0.07 ± 0.01 | 0.46 ± 0.06 | 0.05 ± 0.01 | 0.11 ± 0.02 | 0.06 ± 0.01 | 0.47 ± 0.04 | 0.47 ± 0.05 |
| Co | 0.24 ± 0.02 | 0.20 ± 0.01 | 0.55 ± 0.04 | 0.14 ± 0.01 | 0.81 ± 0.11 | 1.19 ± 0.08 | 0.14 ± 0.01 | 0.29 ± 0.02 | 0.28 ± 0.02 | 0.29 ± 0.02 | 0.99 ± 0.07 | 0.22 ± 0.01 | 0.10 ± 0.01 | 0.88 ± 0.04 |
| Cr | 2.26 ± 0.12 | 1.89 ± 0.11 | 2.78 ± 0.09 | 1.28 ± 0.04 | 4.29 ± 0.21 | 6.21 ± 0.14 | 1.28 ± 0.03 | 2.63 ± 0.03 | 4.27 ± 0.15 | 1.80 ± 0.17 | 3.97 ± 0.16 | 1.78 ± 0.11 | 1.55 ± 0.10 | 3.50 ± 0.13 |
| Cu | 1.98 ± 0.23 | 2.15 ± 0.30 | 1.80 ± 0.18 | 1.97 ± 0.21 | 3.96 ± 0.30 | 4.00 ± 0.33 | 1.97 ± 0.19 | 2.62 ± 0.26 | 2.28 ± 0.22 | 8.85 ± 0.48 | 2.82 ± 0.33 | 1.30 ± 0.17 | 0.89 ± 0.13 | 2.17 ± 0.10 |
| Fe | 258 ± 12 | 225 ± 26 | 654 ± 55 | 120 ± 9 | 632 ± 37 | 1904 ± 25 | 120 ± 8 | 182 ± 6 | 292 ± 9 | 375 ± 10 | 1416 ± 21 | 240 ± 8 | 102 ± 7 | 869 ± 31 |
| K | 6576 ± 169 | 6991 ± 40 | 9743 ± 259 | 7207 ± 61 | 9171 ± 97 | 6749 ± 80 | 7207 ± 95 | 7122 ± 63 | 4458 ± 68 | 5900 ± 140 | 11729 ± 99 | 9749 ± 50 | 5586 ± 111 | 11909 ± 205 |
| Li | 0.39 ± 0.04 | 0.36 ± 0.03 | 0.82 ± 0.10 | 0.25 ± 0.04 | 1.00 ± 0.13 | 2.30 ± 0.22 | 0.25 ± 0.05 | 0.53 ± 0.07 | 0.38 ± 0.03 | 0.68 ± 0.05 | 1.08 ± 0.05 | 0.41 ± 0.07 | 0.16 ± 0.03 | 1.62 ± 0.05 |
| Mg | 1224 ± 78 | 1579 ± 56 | 1672 ± 36 | 1669 ± 27 | 1763 ± 150 | 1627 ± 77 | 1669 ± 54 | 898 ± 65 | 704 ± 22 | 1903 ± 75 | 2371 ± 64 | 1259 ± 32 | 840 ± 39 | 2218 ± 88 |
| Mn | 40.1 ± 2.3 | 36.5 ± 4.0 | 122 ± 9 | 25.3 ± 2.3 | 59.1 ± 4.6 | 117 ± 10 | 25.4 ± 3.5 | 44.0 ± 1.3 | 40.9 ± 5.5 | 23.9 ± 1.3 | 160 ± 12 | 51.9 ± 3.4 | 16.1 ± 1.2 | 261 ± 8 |
| Na | 725 ± 30 | 553 ± 8 | 691 ± 17 | 422 ± 35 | 524 ± 26 | 679 ± 35 | 422 ± 20 | 160 ± 9 | 945 ± 7 | 881 ± 45 | 854 ± 30 | 649 ± 29 | 201 ± 22 | 993 ± 41 |
| Ni | 2.11 ± 0.10 | 1.36 ± 0.09 | 2.62 ± 0.08 | 0.68 ± 0.05 | 6.3 ± 0.26 | 4.80 ± 0.11 | 0.68 ± 0.03 | 3.46 ± 0.05 | 7.62 ± 0.34 | 11 ± 0.45 | 3.88 ± 0.21 | 2.26 ± 0.07 | 1.26 ± 0.07 | 4.02 ± 0.20 |
| P | 1464 ± 75 | 1757 ± 28 | 1736 ± 53 | 652 ± 21 | 789 ± 60 | 865 ± 44 | 1367 ± 36 | 1580 ± 29 | 1059 ± 67 | 1625 ± 116 | 2721 ± 165 | 2298 ± 155 | 912 ± 80 | 1014 ± 55 |
| Pb | 0.63 ± 0.05 | 2.07 ± 0.09 | 0.54 ± 0.02 | 0.76 ± 0.08 | 0.97 ± 0.04 | 0.97 ± 0.07 | 0.54 ± 0.10 | 0.81 ± 0.15 | 1.02 ± 0.16 | 0.75 ± 0.11 | 0.70 ± 0.10 | 0.37 ± 0.06 | 3.65 ± 0.07 | 3.65 ± 0.09 |
| Se | 0.09 ± 0.01 | 0.08 ± 0.01 | 0.10 ± 0.01 | 0.07 ± 0.01 | 0.09 ± 0.01 | 0.12 ± 0.01 | 0.07 ± 0.01 | 0.09 ± 0.01 | 0.08 ± 0.01 | 0.09 ± 0.01 | 0.12 ± 0.02 | 0.07 ± 0.01 | 0.06 ± 0.01 | 0.17 ± 0.01 |
| Sr | 10.2 ± 0.9 | 7.38 ± 0.46 | 12.3 ± 1.1 | 6.66 ± 0.25 | 31.1 ± 2.7 | 34.3 ± 2.8 | 6.66 ± 0.34 | 18.5 ± 1.7 | 9.20 ± 0.94 | 8.88 ± 1.20 | 21.4 ± 0.2 | 9.88 ± 1.01 | 5.55 ± 0.78 | 12.4 ± 1.2 |
| Ti | < *LOQ* | < *LOQ* | < *LOQ* | < *LOQ* | < *LOQ* | < *LOQ* | < *LOQ* | < *LOQ* | < *LOQ* | < *LOQ* | < *LOQ* | < *LOQ* | < *LOQs* | < *LOQ* |
| V | 0.65 ± 0.12 | 1.13 ± 0.18 | 2.06 ± 0.09 | 0.57 ± 0.06 | 2.58 ± 0.22 | 5.73 ± 0.35 | 0.57 ± 0.07 | 0.80 ± 0.11 | 0.79 ± 0.08 | 1.52 ± 0.07 | 3.48 ± 0.14 | 0.62 ± 0.04 | 0.30 ± 0.04 | 3.83 ± 0.23 |
| Zn | 91.8 ± 4.4 | 86.6 ± 7.0 | 176 ± 5 | 71.1 ± 6.2 | 141 ± 15 | 94.8 ± 3.9 | 71.5 ± 5.5 | 3.91 ± 0.38 | 204 ± 16 | 37.5 ± 4.7 | 152 ± 9 | 295 ± 11 | 70.4 ± 3.6 | 153 ± 17 |

The Bi and Ti concentrations were below *LOQ*.
